# A Swollen Dilemma: A Case Report of Concurrent Periorbital Cellulitis and Angioedema

**DOI:** 10.7759/cureus.89249

**Published:** 2025-08-02

**Authors:** Vincent Lee, Manuel De La Cruz Seoane, Sohair Angly, Frontela Odalys, Sahar S Abdelmoneim

**Affiliations:** 1 Internal Medicine, Southwest Healthcare, Palmdale, USA; 2 Internal Medicine, Larkin Community Hospital Palm Springs Campus, Hialeah, USA; 3 General Internal Medicine/Cardiovascular Medicine, Assiut University Hospital, Assiut, EGY

**Keywords:** angioedema, periorbital cellulitis, periorbital edema, periorbital erythema, periorbital pain, periorbital swelling

## Abstract

Periorbital cellulitis and angioedema can present with similar clinical features, posing a diagnostic challenge. We describe a case of a 71-year-old female patient with a past history of hypertension and diabetes who developed facial swelling and erythema following trauma. She was initially treated for periorbital cellulitis but was later found to have concurrent angioedema, likely triggered by antibiotics and herbal supplements. This report emphasizes the importance of thorough clinical evaluation, timely imaging, and consideration of alternative diagnoses to guide appropriate management.

## Introduction

Periorbital cellulitis, also referred to as preseptal cellulitis, is a common infection of the eyelid and surrounding skin anterior to the orbital septum, often resulting from local trauma, sinusitis, or contiguous spread from adjacent skin infections. It is typically caused by bacterial pathogens such as *Staphylococcus aureus* and *Streptococcus* species [1]. Although most cases are uncomplicated and respond well to empiric antibiotic therapy, distinguishing periorbital cellulitis from the more dangerous orbital cellulitis (where the infection spreads posterior to the septum) is essential due to the latter's potential for serious complications such as vision loss, intracranial extension, and cavernous sinus thrombosis. While periorbital cellulitis is more frequently reported in children, adults--particularly those with risk factors such as diabetes, immunosuppression, or recent facial trauma--are also susceptible and may experience more complex clinical courses [2].

In contrast, angioedema is a non-infectious hypersensitivity reaction characterized by localized, rapid swelling of the deeper layers of the skin and mucosa due to increased vascular permeability [3]. It may result from allergic reactions, medication side effects (e.g., ACE inhibitors), or hereditary conditions. Angioedema can affect the face, lips, tongue, and airway, posing life-threatening risks if not promptly managed. It is often idiopathic but can be triggered by drugs, including NSAIDs, antibiotics, or even herbal supplements, as was suspected in this case. Epidemiologically, angioedema affects all age groups but may be more severe in older adults and those with comorbidities like hypertension or diabetes [4].

While each of these conditions is frequently encountered in clinical practice, their simultaneous occurrence is rare and presents a unique diagnostic challenge. The coexistence of periorbital cellulitis and angioedema can confound clinical judgment, as both conditions manifest with facial swelling, erythema, and discomfort. However, their divergent etiologies-infectious versus allergic-require different management strategies. A history of facial trauma may predispose to cellulitis, while the introduction of a new drug or supplement could trigger angioedema, as previously reported [1,3]. More importantly, the rapid progression of facial and periorbital swelling unresponsive to initial antibiotic therapy should raise suspicion for a co-diagnosis.

The case presented here describes a 71-year-old female patient with a history of diabetes and hypertension who developed significant facial and periorbital swelling following a minor facial trauma and subsequent use of herbal medications. Her presentation was initially consistent with periorbital cellulitis, but the later onset of lip and neck swelling, along with airway compromise, pointed to concurrent angioedema. While the presence of Staphylococcal species is often implicated in bacterial cellulitis, no such findings were reported in this case, highlighting the importance of integrating clinical, laboratory, and imaging data for accurate diagnosis.

To our knowledge, reports of simultaneous periorbital cellulitis and angioedema are scarce, particularly in elderly patients with underlying comorbidities, making this case a rare and clinically significant contribution to the literature. This case underscores the critical need for heightened clinical vigilance and a multidisciplinary approach to manage overlapping infectious and allergic conditions effectively. Recognition of potential triggers, such as trauma or new substances (herbal or pharmacologic), and early use of imaging and laboratory evaluation are vital for distinguishing between these entities and initiating appropriate treatment to prevent serious complications.

## Case presentation

A 71-year-old female patient with a history of hypertension and type 2 diabetes mellitus presented to the emergency department with left-sided facial pain, erythema, and marked swelling. She had sustained facial trauma eight days prior after falling and striking her face against a refrigerator. Initially asymptomatic, she began to develop progressive facial swelling and severe localized pain five days post-injury, prompting evaluation. The pain was non-radiating and not exacerbated by ocular movements, rated 10/10 in intensity. She denied fever or visual changes but endorsed chills and malaise. She also reported taking azithromycin and a Medrol dose pack prescribed by her primary care provider two days prior for flu-like symptoms, along with ingesting an unspecified herbal supplement, which may have triggered a hypersensitivity reaction. Although the patient was unable to provide the name or packaging of the supplement, the clinical team attempted to identify it through history-taking and emphasized it as a potential contributing factor.

Her home medications included amlodipine 10 mg at bedtime, glipizide 10 mg twice daily, losartan 100 mg daily, metformin 1000 mg daily, and Tradjenta® (linagliptin) 5 mg at bedtime; losartan, in particular, has been associated with angioedema in some patients. Blood pressure on arrival was 178/68 mmHg; although hypertensive, this was consistent with her baseline given suboptimal chronic control. No history of prior angioedema, allergic reactions, or periorbital infections was reported, which is pertinent to evaluating the unusual nature of this presentation.

**Figure 1:**
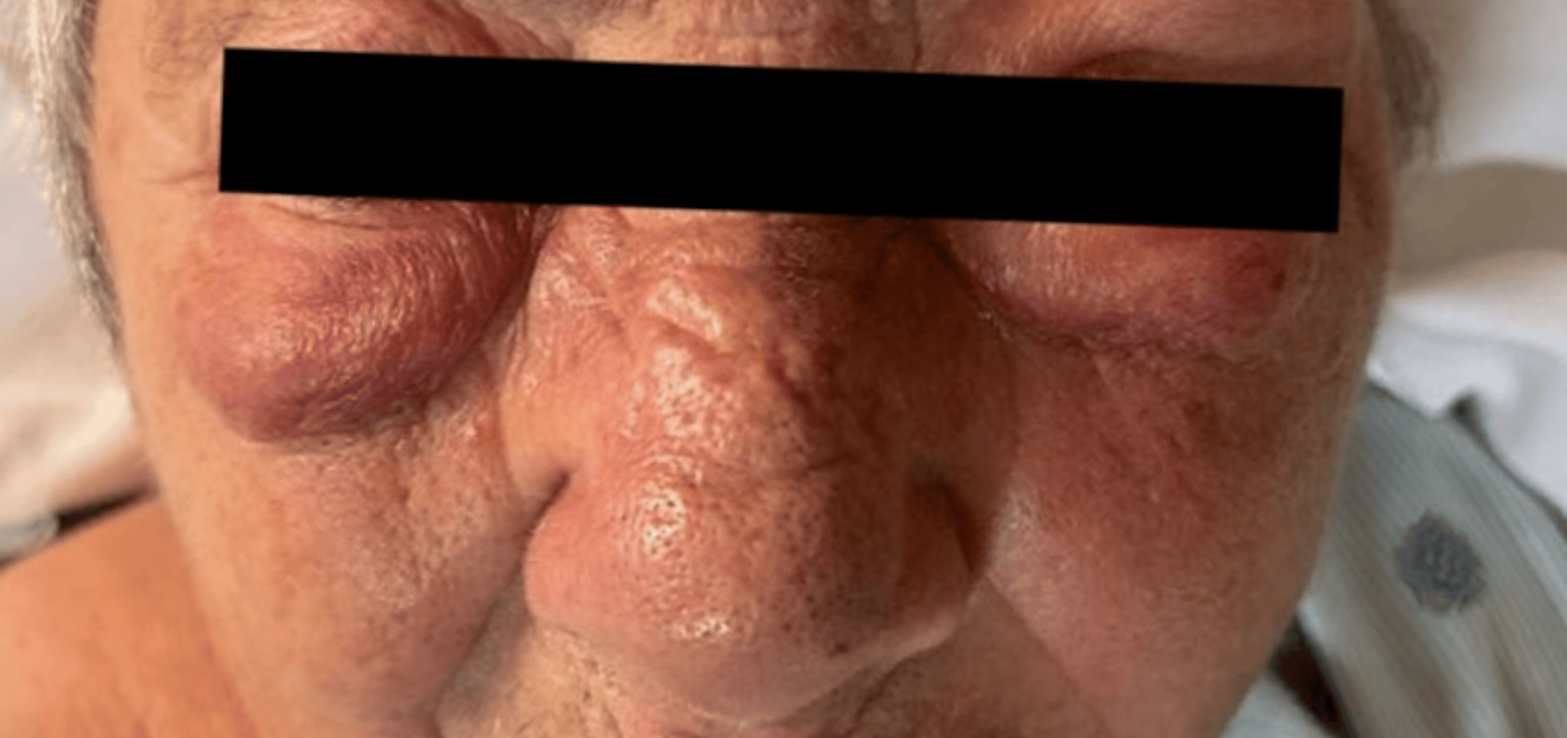
Erythematous periorbital inflammation occluding both eyes with hard, swollen left cheek that are warm to the touch.

On physical exam, the patient appeared in acute distress with left periorbital edema, infraorbital swelling, and erythema, with extension to the left cheek and neck (Figure 1). The eyes were completely occluded, and she exhibited mild tongue swelling and dysphonia. Notably, the swelling later progressed to involve the lips, significantly impairing her ability to speak and raising concerns for airway compromise. She was promptly transferred to the intensive care unit for airway monitoring, where she remained under close observation with continuous pulse oximetry and vital sign assessments every two hours. Bedside airway equipment was readily available, but the patient remained able to protect her airway throughout and did not require intubation.

Initial labs revealed leukocytosis with a WBC count of 23.5 ×10⁹/L, neutrophil predominance (84.2%), thrombocytosis (platelets 458 ×10⁹/L), hypo-osmolarity (260.5 mOsm/kg), and elevated inflammatory markers including CRP >15 mg/L and ESR 71 mm/hr. Serum glucose was persistently elevated (range 196-280 mg/dL), with fasting labs revealing glucosuria (250 mg/dL) and proteinuria. Details of lab values during hospital admission are shown in Table 1. Although she had no continuous glucose monitor (CGM) or recent HbA1c documented, she required insulin titration during hospitalization: insulin glargine was increased to 20 units nightly, and insulin lispro was adjusted to 10 units before meals. The rationale for frequent point-of-care Accu-Chek glucose testing was to closely monitor acute glycemic excursions due to steroid therapy and infection.

**Table 1:** Laboratory data during admission and hospital stay.

Parameter	11/18/2023	11/17/2023	11/16/2023	11/15/2023	11/14/2023	Reference Range
Sodium	133 mmol/L	136 mmol/L	133 mmol/L	135 mmol/L	132 mmol/L	135 - 145 mmol/L
Potassium	4.0 mmol/L	5.4 mmol/L	3.3 mmol/L	3.5 mmol/L	3.9 mmol/L	3.5 - 5.1 mmol/L
Chloride	97 mmol/L	101 mmol/L	97 mmol/L	98 mmol/L	99 mmol/L	96 - 106 mmol/L
Bicarbonate	25 mmol/L	26 mmol/L	25 mmol/L	26 mmol/L	23 mmol/L	22 - 28 mmol/L
Blood urea nitrogen	22 mg/dL	29 mg/dL	27 mg/dL	17 mg/dL	10 mg/dL	7 - 20 mg/dL
Creatinine	0.83 mg/dL	0.90 mg/dL	0.89 mg/dL	0.77 mg/dL	0.66 mg/dL	0.60 - 1.20 mg/dL
Glucose	223 mg/dL	251 mg/dL	226 mg/dL	205 mg/dL	206 mg/dL	70 - 140 mg/dL
Calcium	9.2 mg/dL	9.3 mg/dL	9.3 mg/dL	9.4 mg/dL	8.8 mg/dL	8.5 - 10.2 mg/dL
Albumin	3.6 g/dL	3.4 g/dL	3.4 g/dL	3.5 g/dL	3.8 g/dL	3.5 - 5.0 g/dL
Total protein	6.7 g/dL	6.8 g/dL	6.9 g/dL	7.1 g/dL	7.5 g/dL	6.0 - 8.3 g/dL
Alkaline phosphatase	72 U/L	60 U/L	70 U/L	71 U/L	72 U/L	44 - 147 U/L
Alanine aminotransferase	45 U/L	32 U/L	31 U/L	25 U/L	25 U/L	7 - 56 U/L
Aspartate aminotransferase	47 U/L	35 U/L	31 U/L	31 U/L	21 U/L	10 - 40 U/L
Total bilirubin	0.6 mg/dL	0.6 mg/dL	0.5 mg/dL	0.5 mg/dL	0.8 mg/dL	0.1 - 1.2 mg/dL
Thyroid-stimulating hormone	N/A	N/A	N/A	N/A	0.42 µIU/mL	0.4 - 4.0 µIU/mL
Serum osmolality	267.6 mOsm/kg	277.3 mOsm/kg	269.6 mOsm/kg	268.6 mOsm/kg	260.5 mOsm/kg	275 - 295 mOsm/kg
Lactate	N/A	N/A	N/A	N/A	1.8 mmol/L	<2.2 mmol/L
C-reactive protein - high sensitivity	N/A	N/A	N/A	N/A	>15 mg/L	<1.0 mg/L
White blood cell	16.5 x 10^3^/µL	12.9 x 10^3^/µL	17.9 x 10^3^/µL	22.0 x 10^3^/µL	23.5 x 10^3^/µL	4.0 - 11.0 x 10^3^/µL
Red blood cell	4.60 x 10^3^/µL	4.23 x 10^3^/µL	4.23 x 10^3^/µL	4.25 x 10^3^/µL	4.60 x 10^3^/µL	4.2 - 5.4 x 10^3^/µL
Hemoglobin	14.4 g/dL	13.3 g/dL	13.3 g/dL	13.5 g/dL	14.6 g/dL	12.0 - 16.0 g/dL
Hematocrit	41.7 %	38.5 %	37.7 %	37.7 %	41.0 %	37 - 47 %
Platelet	614 x 10^3^/µL	532 x 10^3^/µL	495 x 10^3^/µL	423 x 10^3^/µL	458 x 10^3^/µL	150 - 450 x 10^3^/µL
Sedimentation rate	N/A	N/A	N/A	N/A	71 mm/hr	0 - 30 mm/hr
Prothrombin time	N/A	N/A	N/A	N/A	10.5 sec	10 - 13 sec
Partial thromboplastin time	N/A	N/A	N/A	N/A	29 sec	25 - 35 sec
International normalized ratio	N/A	N/A	N/A	N/A	0.94	0.80 - 1.20
Mycoplasma	N/A	N/A	N/A	N/A	Negative	Negative
Legionella	N/A	N/A	N/A	N/A	Negative	Negative
Flu	N/A	N/A	N/A	N/A	Negative	Negative
COVID	N/A	N/A	N/A	N/A	Negative	Negative

Initial treatment included intravenous vancomycin and Unasyn® (ampicillin sodium/ sulbactam sodium) 1.5 g every six hours. Procalcitonin was low (0.1 ng/mL), suggesting a limited systemic bacterial infection. By day 2, due to progression of swelling and concern for angioedema, she was treated with intravenous methylprednisolone 60 mg every 8 hours, diphenhydramine 50 mg every 6 hours, and famotidine 40 mg daily. Losartan, a known precipitant of angioedema, was discontinued.

**Figure 2:**
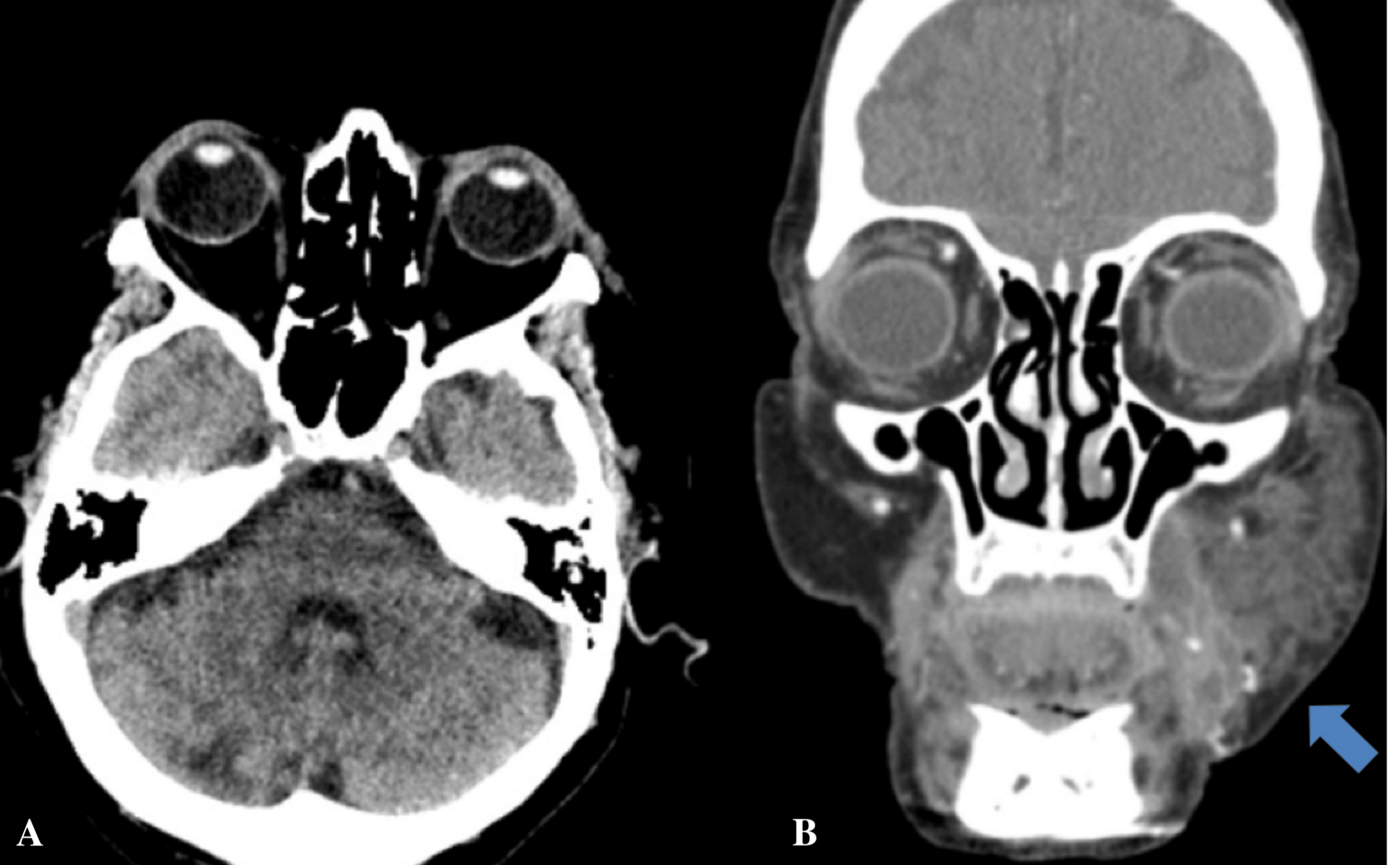
Maxillofacial CT scan - (A) axial section and (B) coronal section revealing soft tissue edema/stranding adjacent to left masticator space (blue) and unremarkable periorbital regions.

Imaging, including non-contrast CT of the maxillofacial region, revealed soft tissue stranding and edema adjacent to the left masticator space without evidence of abscess or orbital involvement (Figure 2). CT brain, cervical spine, chest, and abdomen/pelvis were negative for acute pathology, though a subcentimeter apical pulmonary nodule and incidental nephrolithiasis were noted.

Despite the initial empiric antibiotic regimen, antibiotics were briefly held on infectious disease consultation to reassess need. Given persistent leukocytosis, elevated ESR/CRP, and local signs of infection, antibiotic therapy was escalated on day 3 to intravenous linezolid 600 mg every 12 hours and levofloxacin 750 mg daily, with a favorable response. Blood cultures remained negative, and a urine culture was inconclusive due to mixed flora. No organism, including Staphylococcus aureus, was isolated during admission.

**Figure 3:**
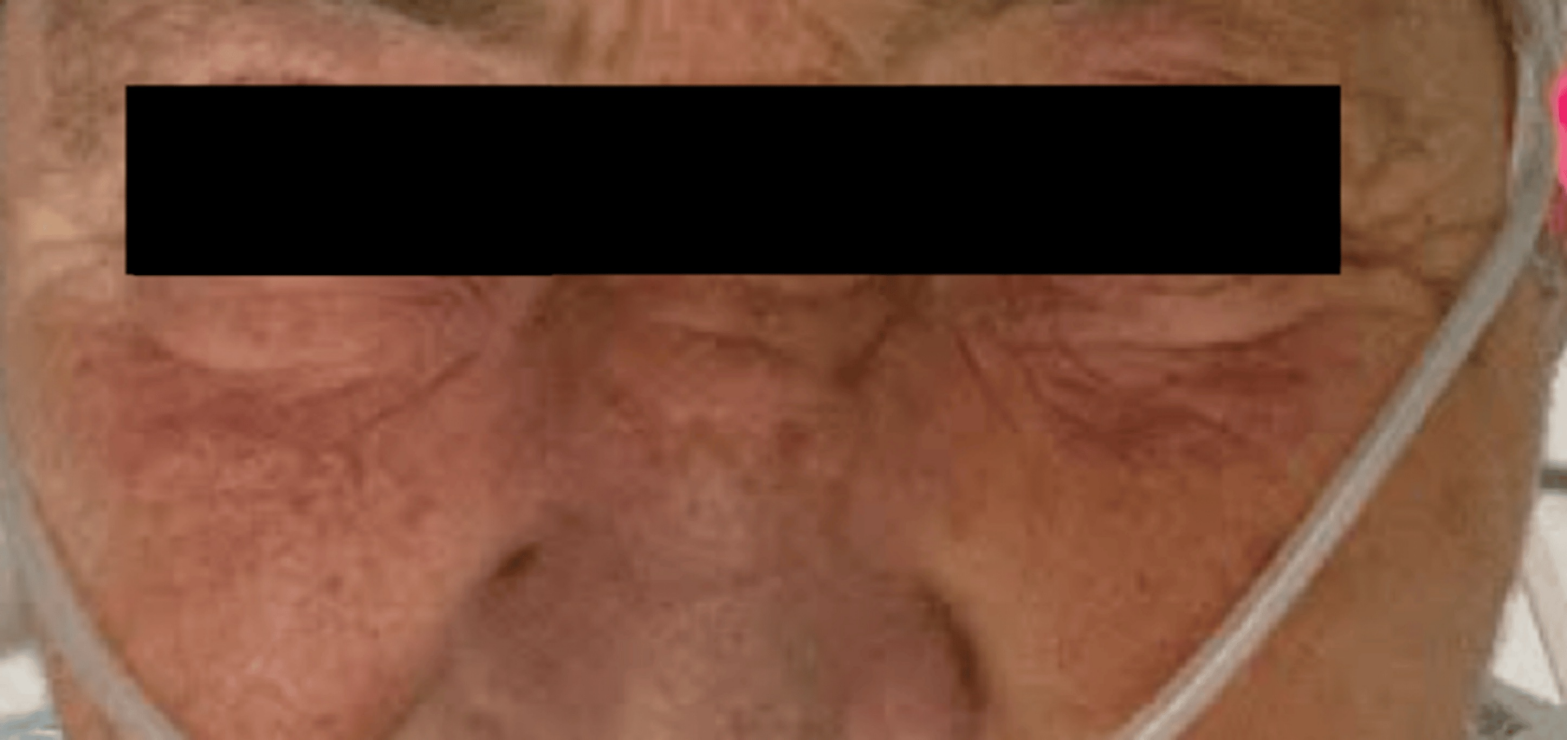
Image showing pre-hospital discharge and complete resolution of the bilateral eye swelling.

The patient stabilized over 72 hours, with substantial reduction in facial and periorbital edema, resumption of oral intake, and improved glycemic control (Figure 3). Although blood and wound cultures were negative, she was discharged on levofloxacin 750 mg daily and doxycycline 100 mg twice daily for 7 days to complete a course of empiric therapy for presumed polymicrobial cellulitis, given her initial clinical presentation and comorbidities. Discharge instructions included strict avoidance of the suspected herbal agent implicated in her angioedema, resumption of home antihypertensive and diabetic medications, outpatient follow-up with primary care, and re-evaluation of the pulmonary nodule per imaging recommendation.

## Discussion

Distinguishing between periorbital cellulitis and angioedema is crucial, as they require markedly different management approaches. While both conditions can present with acute facial swelling, erythema, and discomfort, periorbital cellulitis typically responds to antibiotics targeting bacterial pathogens [1], whereas angioedema necessitates prompt administration of corticosteroids, antihistamines, and often discontinuation of the inciting agent [3]. Misdiagnosis can delay appropriate treatment and potentially endanger the patient’s airway or vision, underscoring the importance of a detailed history and comprehensive clinical evaluation.

The patient’s use of a Medrol dose pack, azithromycin, and an unspecified herbal supplement-all temporally related to the onset of her symptoms-added a layer of diagnostic ambiguity. While her initial presentation (facial trauma, erythema, localized pain, leukocytosis, and elevated CRP) suggested periorbital cellulitis, the later development of lip and tongue swelling with impaired speech and airway concerns pointed toward angioedema, potentially triggered by the herbal product or losartan, which was appropriately discontinued.

Table 2 provides a concise review of previously reported cases where angioedema was misdiagnosed as periorbital cellulitis or vice versa. In the case by Yavuz et al. (2024), a 4-year-old initially diagnosed with angioedema was later found to have viral periorbital cellulitis secondary to Epstein-Barr virus [5]. Gül et al. (2016) described a 14-year-old with angioedema misattributed to anthrax-related periorbital cellulitis [6]. Lucerna et al. (2015) reported a 21-year-old with presumed angioedema who ultimately required incision and drainage for methicillin-resistant Staphylococcus aureus (MRSA) infection [7]. These cases emphasize that even in young patients, overlapping signs can obscure the correct diagnosis and delay targeted therapy.

**Table 2:** Selected studies highlighting reported cases where angioedema was misdiagnosed as cellulitis.

Study	Patient Age	Initial Diagnosis	Corrected Diagnosis	Diagnostic Modality	Treatment Response
Yavuz et al., 2024 [5]	4	Angioedema	Viral cellulitis	CT scan of the head + labs (complete blood count, venous blood gas, chemistry panel, serum C4 and C1 inhibitor esterase, urine electrolytes, urine albumin/creatinine tests)	Steroid and antihistamine
Gül et al., 2016 [6]	14	Angioedema	Periorbital cellulitis	Clinical	Antibiotic and steroid
Lucerna et al., 2014 [7]	21	Angioedema	Cellulitis	CT scan of the head + labs (complete blood count, bacterial culture)	Incision and drainage with antibiotic

Compared to the prior literature, our case is unique for several reasons. First, the patient is an elderly female with comorbid diabetes and hypertension, placing her at greater risk for both infectious and inflammatory complications. Second, her condition followed a minor facial trauma, introducing a mechanical portal for infection, but the progressive involvement of mucosal structures such as the lips and tongue suggested an allergic process. Third, our patient’s care involved a multidisciplinary approach, including infectious disease and critical care teams. Imaging confirmed soft tissue edema without abscess formation, and laboratory studies revealed marked leukocytosis, thrombocytosis, hypo-osmolarity, and elevated inflammatory markers (CRP and ESR), all of which supported an infectious-inflammatory process. However, low procalcitonin and lack of identifiable pathogens, even after culturing, tempered the strength of a purely bacterial hypothesis.

Our treatment course aligned with current best practices but required escalation and adaptation based on evolving clinical signs. Initial use of vancomycin and Unasyn® (ampicillin sodium/ sulbactam sodium) was appropriate for presumed cellulitis [8], and the subsequent shift to corticosteroids and antihistamines upon suspicion of angioedema was timely and likely prevented further respiratory compromise [9]. Upon further diagnostic clarification and interdisciplinary review, the antibiotic regimen was revised to include linezolid and levofloxacin, and the patient ultimately improved without surgical intervention. Fresh frozen plasma (FFP), which may be considered in refractory or hereditary angioedema, was not required in this case, given rapid resolution with conventional therapy [10].

In summary, this case reinforces the importance of maintaining a broad differential diagnosis in facial swelling, particularly when initial treatments do not yield expected improvement. Clinicians should consider co-existing diagnoses, such as cellulitis with superimposed angioedema-when signs evolve atypically. Diagnostic tools, including laboratory markers, imaging, and close patient monitoring, are essential, but clinical judgment based on history and progression remains paramount. Future studies might further examine outcomes in patients with dual diagnoses to help develop optimized diagnostic algorithms and treatment protocols.

## Conclusions

Clinicians should maintain a high index of suspicion for angioedema in patients with worsening facial swelling despite antibiotic therapy. A systematic approach, including imaging, medication review, and response to therapy, can aid in distinguishing between periorbital cellulitis and angioedema, ensuring appropriate management and preventing unnecessary interventions.
